# Integration of bulk/scRNA-seq and multiple machine learning algorithms identifies PIM1 as a biomarker associated with cuproptosis and ferroptosis in abdominal aortic aneurysm

**DOI:** 10.3389/fimmu.2024.1486209

**Published:** 2024-12-11

**Authors:** Zonglin Han, Xiulian Lu, Yuxiang He, Tangshan Zhang, Zhengtong Zhou, Jingyong Zhang, Hua Zhou

**Affiliations:** ^1^ Department of Vascular Surgery, Shandong Provincial Hospital Affiliated to Shandong First Medical University, Jinan, Shandong, China; ^2^ Cisen Pharmaceutical Co., Ltd, Jining, Shandong, China; ^3^ Department of Vascular Surgery, Jinan Seventh People’s Hospital, Jinan, Shandong, China; ^4^ Department of Vascular Surgery, The First Affiliated Hospital of Shandong First Medical University, Jinan, Shandong, China

**Keywords:** PIM1, ferroptosis, cuproptosis, abdominal aortic aneurysm, WGCNA

## Abstract

**Background:**

Abdominal aortic aneurysm (AAA) is a serious life-threatening vascular disease, and its ferroptosis/cuproptosis markers have not yet been characterized. This study was aiming to identify markers associated with ferroptosis/cuproptosis in AAA by bioinformatics analysis combined with machine learning models and to perform experimental validation.

**Methods:**

This study used three scRNA-seq datasets from different mouse models and a human PBMC bulk RNA-seq dataset. Candidate genes were identified by integrated analysis of scRNA-seq, cell communication analysis, monocle pseudo-time analysis, and hdWGCNA analysis. Four machine learning algorithms, LASSO, REF, RF and SVM, were used to construct a prediction model for the PBMC dataset, the above results were comprehensively analyzed, and the targets were confirmed by RT-qPCR.

**Results:**

scRNA-seq analysis showed Mo/MF as the most sensitive cell type to AAA, and 34 cuproptosis associated ferroptosis genes were obtained. Pseudo-time series analysis, hdWGCNA and machine learning prediction model construction were performed on these genes. Subsequent comparison of the above results showed that only PIM1 appeared in all algorithms. RT-qPCR and western blot results were consistent with sequencing results, showing that PIM1 was significantly upregulated in AAA.

**Conclusion:**

In a conclusion, PIM1 as a novel biomarker associated with cuproptosis/ferroptosis in AAA was highlighted.

## 1 Introduction

Abdominal aortic aneurysm (AAA) is a potentially fatal disease characterized by high morbidity and mortality in the event of aortic rupture. In the past 20 years, there have been major advances in surgical and endovascular repair techniques for AAA. However, drug-based treatments are still scarce, highlighting a real need for a deeper understanding of the molecular mechanisms involved in AAA formation (1). The main pathological features of AAA include capillary smooth muscle degeneration and death, of note, the accumulation of inflammatory cells is also an important feature (2, 3). The inflammatory process plays an integral role in AAA and exerts influence on many determinants of aortic wall remodeling.

Monocytes-macrophages include promonocytes in the bone marrow, monocytes in peripheral blood, and macrophages (MF) in tissues. MF are derived from monocytes in the blood, and monocytes are derived from precursor cells in the bone marrow (2, 4, 5). A deeper understanding of monocytes in the peripheral blood and MF will help improve our understanding of the role of these cells in the immune environment of the disease and make it possible to design new macrophage-targeted therapies (6). However, this part of the research is still not very clear in AAA so far.

Metal ions are important mediators of cellular processes, like regulation of cell death, and their abnormal concentration levels can cause destructive effects in cells. Ferroptosis is an iron-dependent programmed cell death. Excessive accumulation of intracellular iron leads to the accumulation of lipid peroxides, which induce programmed cell death (7, 8). Disorders of iron metabolism are closely related to AAA, leading to extensive research on iron-dependent toxicity (9–11). Like iron ions, copper ions also play a role in cell death. High levels of intracellular copper can cause cytotoxicity, also known as cuproptosis (12, 13). This death mechanism is controlled by copper ions and caused by their direct participation in the tricarboxylic acid cycle pathway, leading to protein aggregation and proteotoxic stress response (13). Less studies have reported the potential role of cuproptosis in the prediction of AAA (14). Moreover, the mechanisms behind cuproptosis-associated AAA and its potential link to ferroptosis remain the subject of ongoing investigation.

Here, in this study, we aimed to identify biomarkers based on cuprotosis-related ferroptosis genes in AAA. Comprehensive bioinformatics analysis and machine learning models found that among cuprotosis-related ferroptosis genes, PIM1 (Pim-1 proto-oncogene, serine/threonine kinase) was significantly upregulated in all *in vitro* models and patient PBMCs. In addition, our experimental results confirmed that the gene and protein expression levels of PIM1 were also in a significant upregulation trend. Our findings may help improve the personalized treatment and prognosis assessment of AAA.

## 2 Methods and materials

### 2.1 Data acquisition

In this study, we collected mouse single-cell sequencing data from three different AAA models, including CaCl_2_-induced (GSE164678), elastase-induced (GSE152583), and Ang II-induced (GSE221789) models (15–17). We obtained PBMC bulk RNA transcriptome data from seven patients and seven donors (18). After merging and removing abnormal samples, 13 samples were finally included for analysis. Detailed sample collection information is shown in **Supplementary Table S1**.

### 2.2 Data processing workflow

For single-cell datasets, we used the widely validated Seurat’s (R package, V4.4.0) canonical correlation analysis (CCA) integrated dimensionality reduction pipeline (19). First, we filtered the individual data with a filtering threshold of nFeature_RNA > 200, 50000 > nCount_RNA > 1000 and percent.mt < 10. Next, we used the *FindIntegrationAnchors* function to identify anchors between different datasets and used the I*ntegrateData* function for CCA integration. Subsequently, the top 20 PCs were used for UMAP dimensionality reduction and resolution = 0.3 was used for cluster identification. For cell type identification, we used classical marker genes for different cell types.

For PBMC bulk RNA transcriptome data, we used the principal components analysis (PCA) analysis of DESeq2 (R package, V1.42.1) to filter samples, and we eliminated one AAA sample because the characteristics of this sample were consistent with those of the donor (20).

### 2.3 Cell sensitivity analysis

To identify the cell types most affected by AAA, we performed a cell sensitivity analysis using Augur (R package, V1.0.0). The AUC feature value was calculated using *calculate_auc* function, with the input being the constructed Seurat object. The codes refer to https://github.com/neurorestore/Augur.

### 2.4 Cell-cell communication analysis

The CellChat package (R package, V1.6.1) was used to construct the intercellular communication network (21). We constructed cellchat objects between the control and AAA samples to better compare and visualize them. The ligand receptor database is provided by the software package.

### 2.5 Identification of differently expressed genes

The schemes for identifying DEGs in single-cell data and bulk RNA-seq data are slightly different. For single-cell data, we used the *FindMarkers* function of the Seurat software package for identification (19), with a selection threshold of |log2FC| > 0.25 and *p* < 0.05. For bulk RNA-seq, the DESeq2 software package was used for identification (20), with a selection threshold of |log2FC| > 0.5 and *p* < 0.05 (22).

### 2.6 Function enrichment analysis

In order to explore the functional status of gene sets and select subsequent signal targets as much as possible, we used clusterProfiler (R package, V4.10.1) to perform functional enrichment analysis, including gene ontology (GO), kyoto encyclopedia of genes and genomes (KEGG), gene set enrichment analysis (GSEA) and Reactome Pathway enrichment (23). Terms with *p* < 0.05 were considered significantly enriched. We used aPEAR (R package, V1.0.0) for some of the visualizations and ggplot2 (R package, V3.5.1) for the rest (24).

### 2.7 Identification of cuproptosis associated ferroptosis genes

To identify the interrelated cuproptosis associated ferroptosis genes in AAA, we obtained ferroptosis genes and cuproptosis from the Ferropoptosis Database (FerrDb, V2, http://www.zhounan.org/ferrdb/current/) (25). Then, we used Hmisc (R package, V5.1-2) to calculate the Pearson correlation between cuproptosis genes and ferroptosis genes. The Pearson correlation coefficient is widely used to measure the degree of correlation between two variables. Its value is between -1 and 1. >0 indicates a positive correlation, and <0 indicates a negative correlation. In this study, we defined |r| > 0.8 as a strong correlation, and we considered |r| > 0.8 and *p* < 0.05 to be a significant correlation, that is, cuproptosis associated ferroptosis genes.

### 2.8 Construction of protein-protein interaction network

To characterize the relationships of cuproptosis genes, we performed PPI analysis and visualization using the STRING (V12.0, https://string-db.org/cgi/input.pl?sessionId=0kGn9YYW1ES0&input_page_active_form=multiple_identifiers) database. The input was all cuproptosis genes.

### 2.9 Construction of pseudo-time developmental trajectories

To investigate transcriptional signatures that regulate cell fate transitions, we performed pseudo-time developmental trajectories construction using monocle (R package, V2.24.0) (26). We first constructed the monocle_cds object of monocytes and macrophages, then used the reduceDimension function for trajectory construction and the orderCells function for cell sorting. Notably, the BEAM statistical analysis model was used for cell fate trajectory differential analysis.

### 2.10 High dimensional weighted gene co-expression network analysis

WGCNA is widely used to identify the relationship between gene modules and phenotypes (27, 28). In high-dimensional data, such as scRNA-seq, it is difficult to achieve due to the characteristics of the data. The development of hdWGCNA (R package, V0.3.03) software makes it possible to run single-cell datasets (29). Our data object is the single-cell expression matrix of monocytes and macrophages. The relevant analysis code refers to the software tutorial (https://smorabit.github.io/hdWGCNA/).

### 2.11 Different machine learning algorithms identify candidate biomarkers

The screening of clinical biomarkers is the foundation for the early stage of clinical application transformation. In order to identify key biomarkers from massive sequencing data, we used four different machine learning algorithms, including least absolute shrinkage and selection operator (LASSO), recursive feature elimination (RFE), random forest (RF) and support vector machine (SVM). Of note, all machine learning methods were tested on PBMC bulk RNA transcriptome data (18).

The LASSO model was established using the cv.*glmnet* function of the glmnet (R package, V4.1-8), and the parameters were set as *type.measure = ‘mse’, nfolds = 5 and alpha = 1*. Then, the minimum lambda value was used as a reference to determine the optimal parameters.

The RFE model was established using the *rfe* function in caret (R package, V6.0-94), with parameters set as *metric = ‘Accuracy’, sizes = 1:(length(candidate.gene)-2).*

*rfeControl = control*, and 10-fold cross validation was used.

The RF model was established using the *randomForest* function in randomForest (R package, V4.7-1.1), with parameters set as *importance=T, proximity=T, ntree= optionTrees.*

The SVM model was built using the *svmRFE* function in e1071 (R package, V1.7-13), with parameters set as *k = 5, halve.above = 100*.

### 2.12 RT-qPCR

RT-qPCR was used to measure gene expression levels in PBMC and the steps were as described in our previous publication (30). In a short, gene was amplified and quantitatively analyzed after reverse transcription. The method of 2^−ΔΔC^ was used to obtain the fold change. GAPDH was the housekeeping gene. The gene primers was in the **Supplementary Table S2**.

### 2.13 Western blot for detecting the expression of protein

Western Blot was used to measure protein expression levels in PBMC and the steps were as described in our previous publication (30). Briefly, cells were lysed with enhanced RIPA lysis buffer and proteins were obtained and then denatured at high temperature. Next, SDS-PAGE electrophoresis and antibody incubation (Abcam, ab300453, the dilution ratio = 1:1000) were performed. Finally, protein chemiluminescence was performed and photos. ImageJ software was selected to calculate protein gray value.

### 2.14 Statistical analysis

Three independent replicates were performed in all biological experiments and the data were visualized as mean ± SD; Student’s t test was used to identify the differences between AAA and normal using Graphpad Prism 8.0 software. * indicates *p* < 0.05, compared with the control group.

## 3 Results

### 3.1 Single-cell landscape of AAA under different models

**Figure 1:**
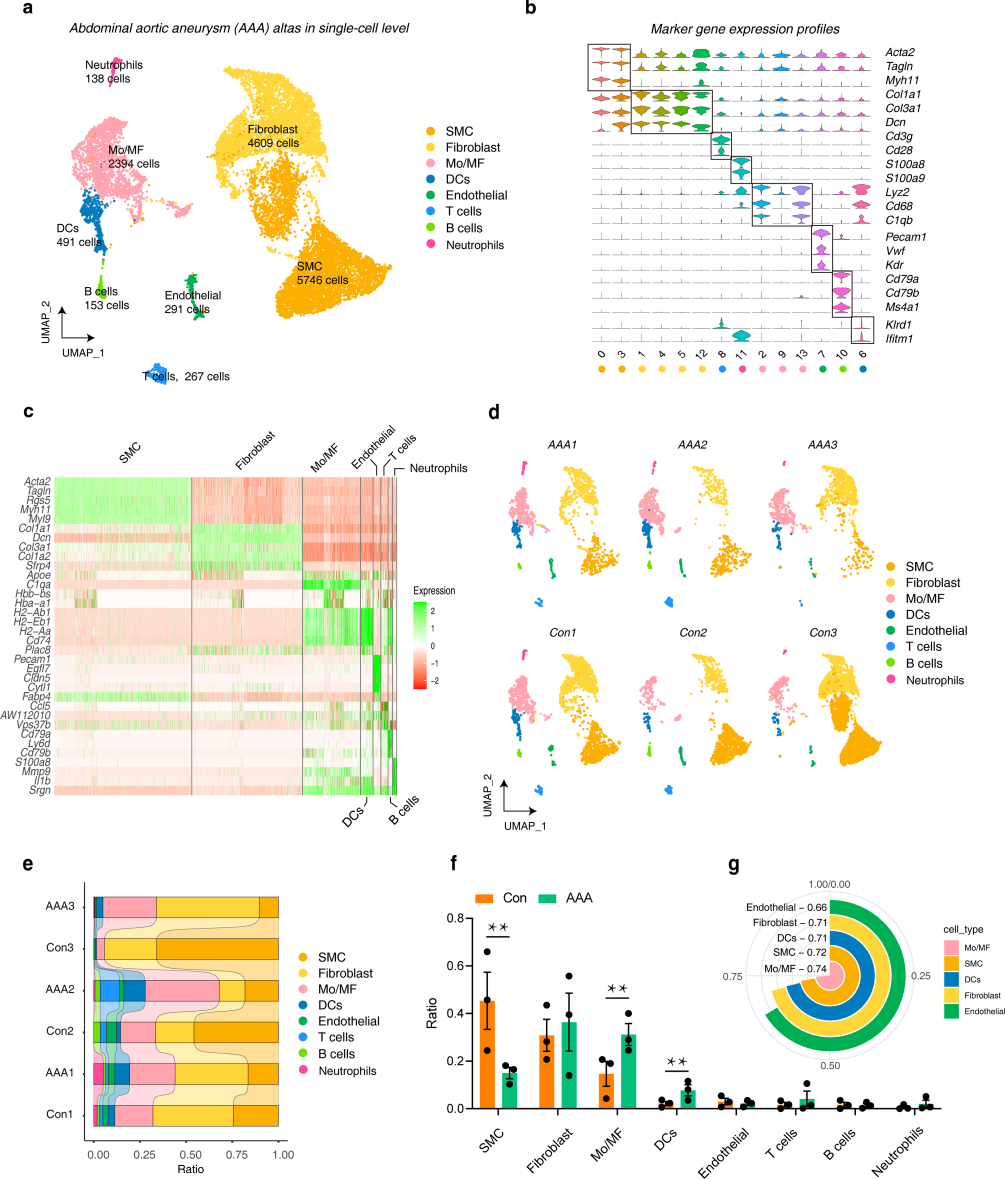
The landscape of AAA in a single-cell resolution. **(A)** UMAP plots show the cell distribution and number of different cell types included in the analysis under the three AAA models. **(B)** Violin plots show the expression characteristics of marker genes in different cell types **(C)** Heatmap showing the top 5 genes in different cell types. **(D)** UMAP plots show the distribution of cell types in different samples. **(E)** The distribution ratio of different cell types in each sample. **(F)** The bar graphs show the distribution statistics of different cell types under AAA and Con. Statistics were performed using Student’s T test, ** represents *p* < 0.01. **(G)** The circular histogram shows the sensitivity of different cell types to AAA, calculated by Augur. AAA1 means CaCl_2_-induced model, AAA2 means elastase-induced model and AAA3 means Ang II-induced model.

We collected three different datasets to study the single-cell landscape in AAA. A series of quality control and integration analyses showed that we integrated samples from different groups well to meet the needs of downstream analysis (**Supplementary Figure S1A**). Next,14,089 cells were divided into 17 cell clusters and annotated into 8 cell types (**Supplementary Figure S1B**), including 5,746 smooth muscle cells (SMCs, marker genes were *Acta2, Tagln* and *Myh11*), 4,609 Fibroblasts (marker genes were *Col1a1, Col3a1 and Dcn*), 2,394 monocytes and macrophages (Mo/MF, marker genes were *Lyz2, Cd68 and C1qb*), 491 dendritic cells (DCs, marker genes were *Col1a1, Col3a1 and Dcn*), 291 Endothelials (marker genes were *Pecam1, Vwf and Kdr*), 267 T cells (marker genes were *Cd3g and Cd28*), 153 B cells (marker genes were *Cd79a, Cd79b and Ms4a1*), and 1,38 Neutrophils (marker genes were *S100a8 and S100a9*) (**Figures 1A, B**; **Supplementary Figure S1C**). We then performed cluster analysis on the expression of top 5 marker genes in different cell types (**Figure 1C**). UMAP was used to display the dimensionality reduction distribution of different cell types in different samples (**Figure 1D**). Subsequently, we observed the proportion of different cell types in different samples, and further used bar charts to visualize the cell types in different groups. The results showed that the proportion of SMC in AAA was significantly reduced, while Mo/MF and DCs were significantly increased in AAA (**Figures 1E, F**). Of note, we used the AUGUR algorithm to calculate cell sensitivity, and the results showed that Mo/MF was the most sensitive to AAA, followed by SMC and DCs (**Figure 1G**). This result was consistent with the statistical proportion of cell types. The above results showed that the proportion of SMCs decreased in AAA, which may be the result of massive apoptosis of SMCs (31). In addition, Mo/MF and DCs increased significantly, which may be related to their participation in inflammatory response (2). Since Mo/MF are the most sensitive cells, we included them in the subsequent analysis.

### 3.2 Cell-cell communication landscape of AAA under different models

**Figure 2:**
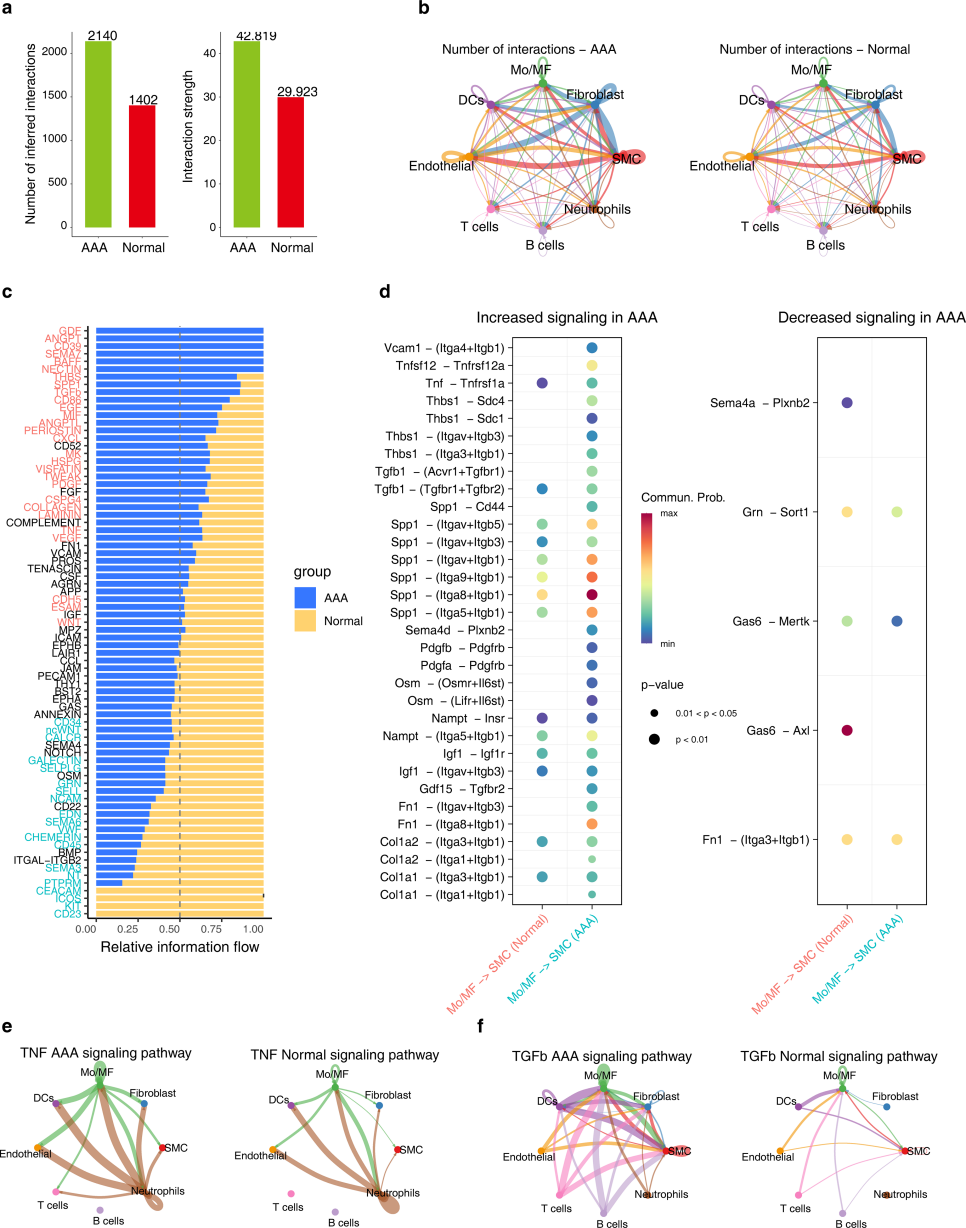
The cellular communication between SMC and Mo/MF is altered in AAA. **(A)** The bar graph shows the communication frequency (left) and strength (right) of all cell types in different groups. **(B)** The network diagram shows the frequency of cell communication for each cell type in different groups. **(C)** The stacked bar chart shows the differences in each signaling pathway under different groups. **(D)** The bubble chart shows the differential communication signals between Mo/MF and SMC in different groups. The left side shows the up-regulated signals in AAA, and the right side shows the down-regulated signals. e and f, Network diagram showing the differences in TNF-α **(E)** and TGF-*β* **(F)** signaling in different groups.

After integrated analysis of different AAA-induced models, it was found that in AAA, the intensity and frequency of intercellular communication were significantly enhanced, especially between SMC, Mo/MF, Fibroblast and Endothelial (**Figures 2A, B**). We then compared the differences in different signaling pathways between AAA and normal groups and found that GDF, ANGPT, and CD39 only appeared in AAA, while CD23, CEACAM, etc. only appeared in the normal group (**Figure 2C**). Since Mo/MF are AAA-sensitive cells and SMC loss is the main pathological feature of AAA, we observed the communication between these two types of cells. The results showed that TNF-α (*Tnf*-*Tnfrsf1a*) and TGF-*β* (*Gdf15*-*Tgfbr*2) signals were significantly enhanced in AAA (**Figure 2D**). There is also strong communication from endothelial cells to Mo/MF. Interestingly, the data showed that upregulation of TNF-α (*Tnf*-*Tnfrsf1a*) and TGF-*β* (*Gdf15*-*Tgfbr*2) signals was also observed in Mo/MF-Endoithelial (**Supplementary Figure S2A**). In addition, we observed the communication between TNF-α and TGF-*β* signals among different cell types throughout the sample, and the results showed that TNF-α and TGF-*β* signaling were overall enhanced in AAA (**Figures 2E, F**).

### 3.3 Transcriptional landscape of Mo/MF under different models

**Figure 3:**
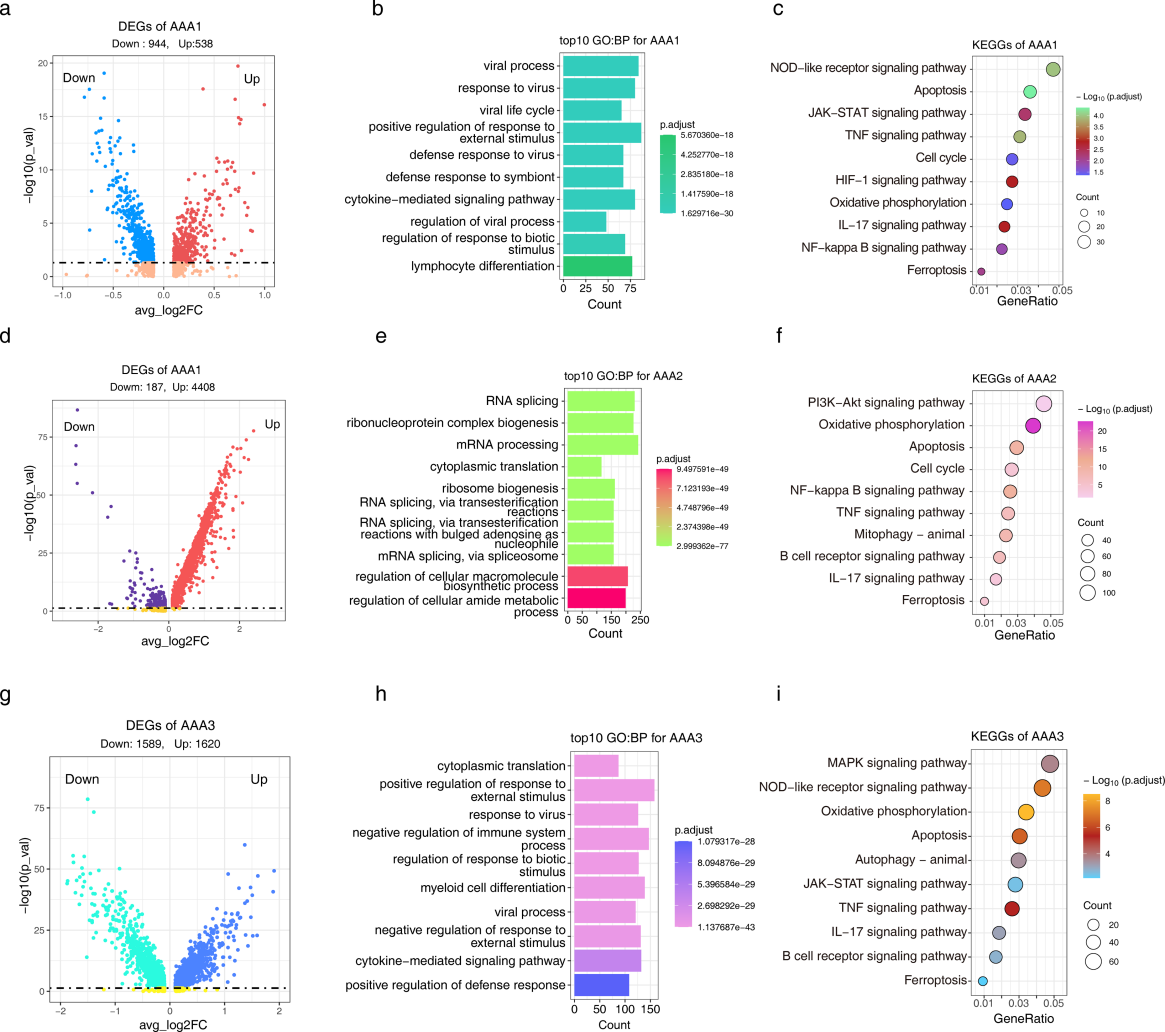
The transcriptional dynamics of Mo/MF were altered under different models. **(A)** The volcano plot shows the DEGs of Mo/MF under CaCl2-induced model, the DEGs screening threshold was |log2FC| > 0.25 and *p* < 0.05. **(B)** The GO enrichment analysis results of DEGs, *p* < 0.05 was considered significantly enriched. **(C)** The KEGG enrichment analysis results of DEGs, *p* < 0.05 was considered significantly enriched. D-F, DEGs **(D)** and related GO **(E)** and KEGG **(F)** enrichment analysis results under the elastase-induced model. **(G–I)**, DEGs **(G)** and related GO **(H)** and KEGG **(I)** enrichment analysis results under the Ang II-induced model.

In the CaCl_2_-induced (GSE164678) model, we obtained a total of 1,482 DEGs, of which 944 were downregulated and 538 were upregulated (**Figure 3A**). GO results showed that these genes may be involved in ‘lymphocyte differentiation’ (**Figure 3B**). KEGG pathways showed that these genes play a role in regulating ‘apoptosis’, ‘TNF-α signaling pathway’, ‘cell cycle’ and ‘ferroptosis’ (**Figure 3C**). For elastase-induced (GSE152583) model, there were 4,595 DEGs, and most of them were upregulated, with 4,408 (**Figure 3D**). The GO annotation of these genes showed that they might be involved in ‘RNA splicing’ and ‘mRNA processing’ (**Figure 3E**), and the KEGG pathway enrichment showed that the ‘PI3K-AKT signaling pathway’, ‘apoptosis’, ‘cell cycle’, ‘TNF-α signaling pathway’, and ‘ferroptosis’ signaling pathway were significantly enriched (**Figure 3F**). For Ang II-induced (GSE221789) model, a total of 3209 DEGs were observed (**Figure 3G**), and their GO results showed that they might be involved in the ‘cytokine-mediated signaling pathway’ (**Figure 3H**). At the same time, the KEGG functional enrichment entries showed enrichment of ‘apoptosis’, ‘TNF-α signaling pathway’, and ‘ferroptosis’ signaling pathway (**Figure 3I**). These results suggest that ferroptosis may be involved in regulating the progression of AAA.

### 3.4 Comparative analysis of Mo/MF under different AAA models

**Figure 4:**
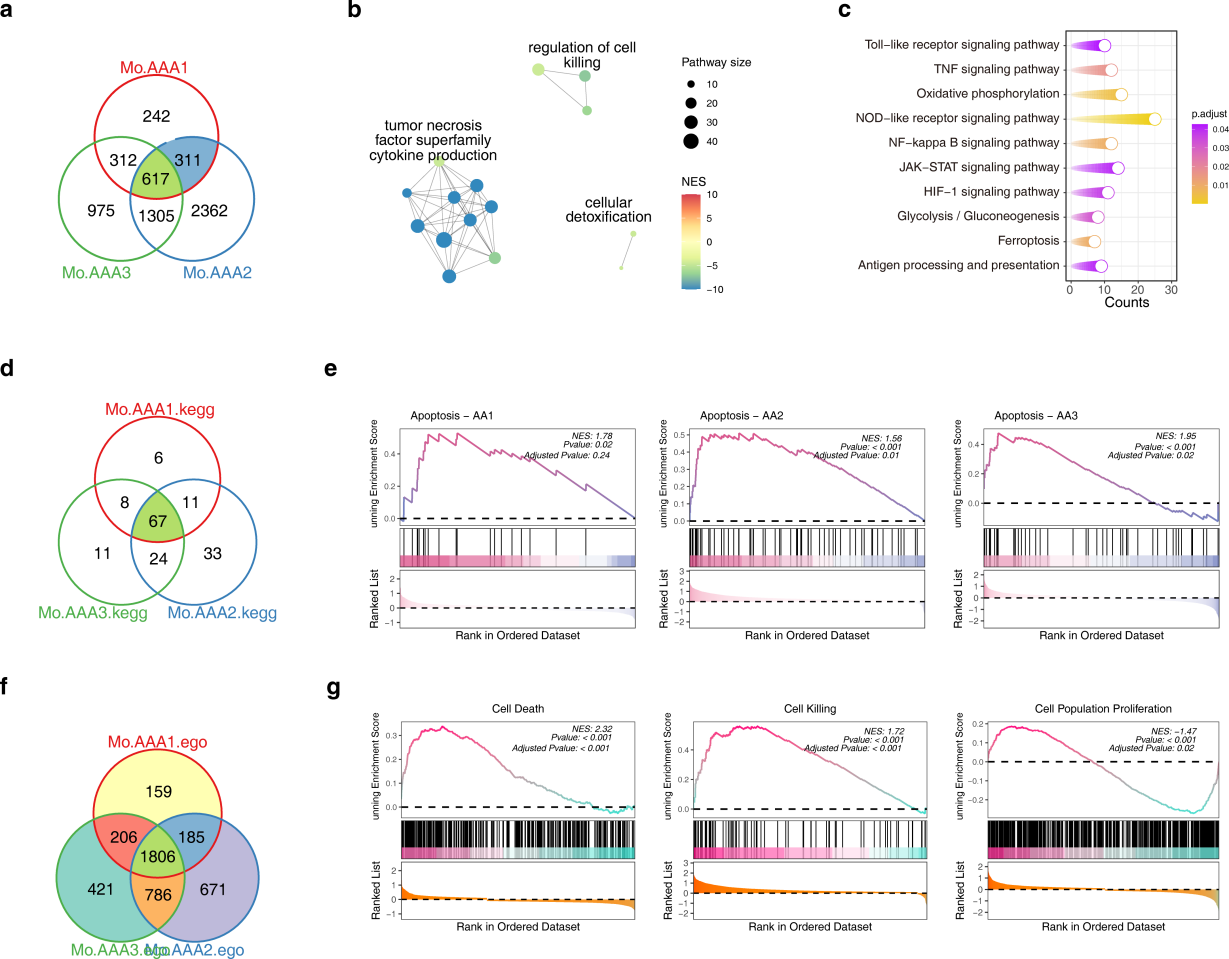
Comparative analysis of Mo/MF differences under different models. **(A)** The Venn diagram showing the comparative analysis of differentially expressed genes in Mo/MF under different models. **(B, C)** The GO **(B)** and KEGG **(C)** enrichment results of common DEGs in different models, *p* < 0.05 was considered significantly enriched. **(D)** The Venn diagram shows the comparative analysis of KEGG pathways in different models. **(E)** GSEA results showed the status of the Apoptosis signaling pathway under different models. **(F)** The Venn diagram shows the comparative analysis of GO terms in different models. **(G)** GSEA results showed the status of the cell death, cell killing and cell population proliferation signaling pathway under different models. AAA1 means CaCl_2_-induced model, AAA2 means elastase-induced model and AAA3 means Ang II-induced model.

To further investigate the transcriptional signatures of Mo/MF under different induction models to better understand the mechanism of AAA, we first compared the differences in DEGs under different models, and the results showed that there were 617 shared genes (**Figure 4A**). The GO enrichment of these genes showed that they might be involved in the ‘TNF-α supurfamily cyttokine production’ (**Figure 4B**). In addition, the functional enrichment results suggested that the ‘TNF-α signaling pathway’ and ‘ferroptosis’ signaling pathway were significantly enriched (**Figure 4C**). Next, we compared the differences in different KEGG signaling pathways, and the results showed that 67 shared signaling pathways were significantly enriched in different models, such as apoptosis, ferroptosis, etc. (**Figure 4D**). Interestingly, GSEA showed that the apoptosis signaling pathway was significantly activated in AAA under different models (**Figure 4E**). Subsequent GO comparative analysis revealed 1,806 common terms (**Figure 4F**), and GSEA showed that cell death signals and cell killing signals were significantly activated in AAA, while cell population proliferation signals were significantly inhibited (**Figure 4G**). These results again highlight the role of ferroptosis signaling in AAA.

### 3.5 Identification of cuprotosis associated ferroptosis genes in Mo/MF

**Figure 5:**
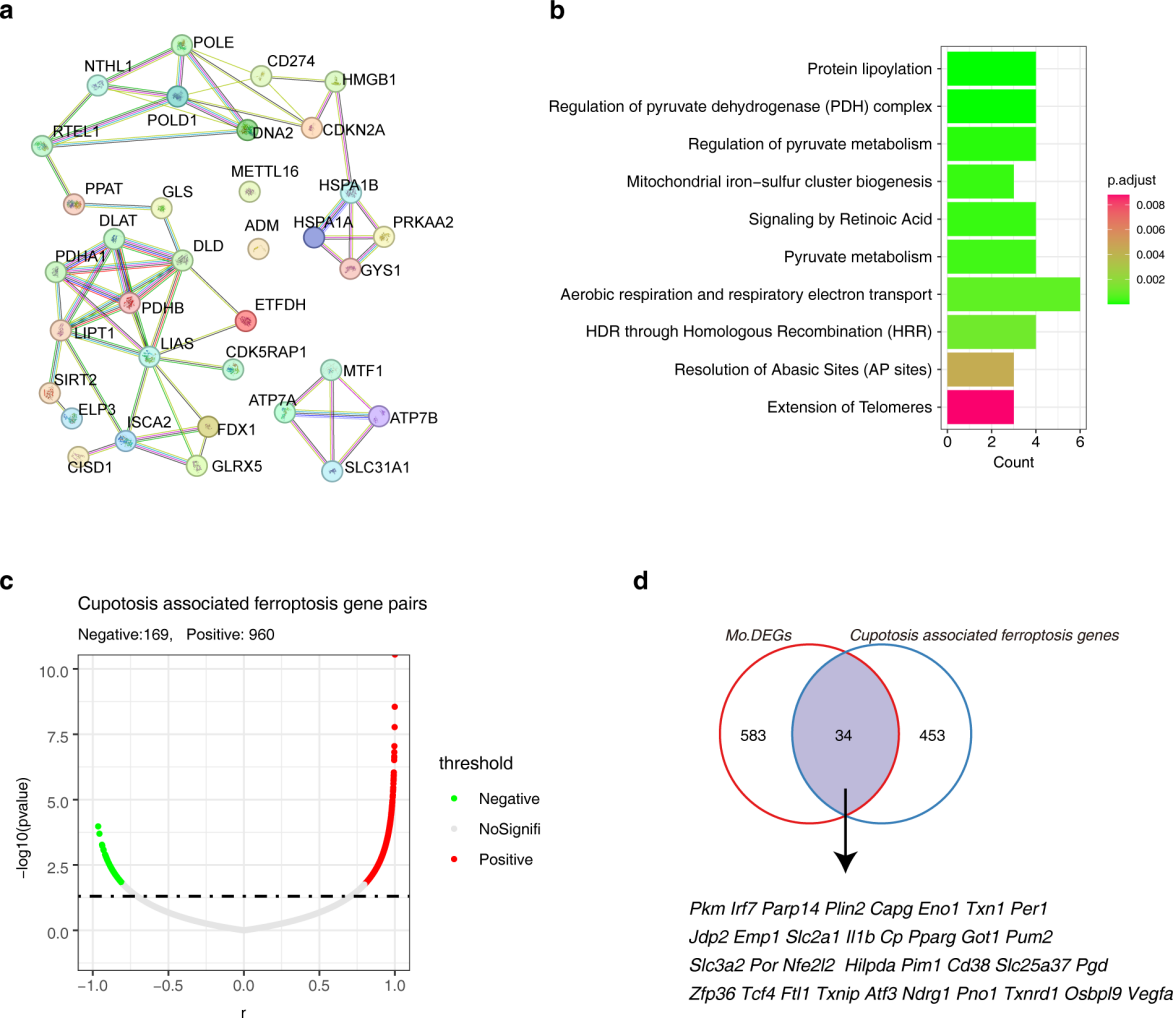
Identification of cuproptosis-associated ferroptosis genes in AAA. **(A)** The PPI network shows the interactions among cuprotosis genes. **(B)** The bar graph shows the Reactome Pathway of cuproptosis genes. **(C)** The volcano plot shows the correlation between candidate ferroptosis genes and cuproptosis genes. **(D)** The Venn diagram showing the relationship between candidate ferroptosis genes and DEGs. AAA1 means CaCl_2_-induced model, AAA2 means elastase-induced model and AAA3 means Ang II-induced model.

Since the above results repeatedly suggested the role of ferroptosis in AAA, and taking the crosstalk between cuprotosis and ferroptosis into consideration, we attempted to identify the cuprotosis associated ferroptosis genes. We first explored the PPI network of cuptosis genes themselves. The results showed that PDHA1, DLAT, DLD, PDHB, LIPT1 and SIRT2 had strong interactions (**Figure 5A**). Reactome pathway enrichment results showed that these cuprotosis genes were involved in ‘protein lipoylation’ and ‘pyruvate metabolism’ (**Figure 5B**). Next, Pearson correlation analysis was performed to obtain the cuprotosis-ferroptosis gene pairs in AAA, among them, there were 169 negatively correlated (r < -0.8, *p*< 0.05) and 960 positively correlated (r > 0.8, *p*< 0.05) gene pairs. Of note, 1129 gene pairs contain 487 cuprotosis-associated ferroptosis genes (**Figure 5C**; **Supplementary Table S3**). To narrow down the candidate range, we compared these 487 cuprotosis-associated ferroptosis genes with the Mo/MF shared DEGs in different models and obtained 34 candidate genes for subsequent analysis (**Figure 5D**).

### 3.6 Developmental trajectories of cuprotosis-associated ferroptosis genes in Mo/MF

**Figure 6:**
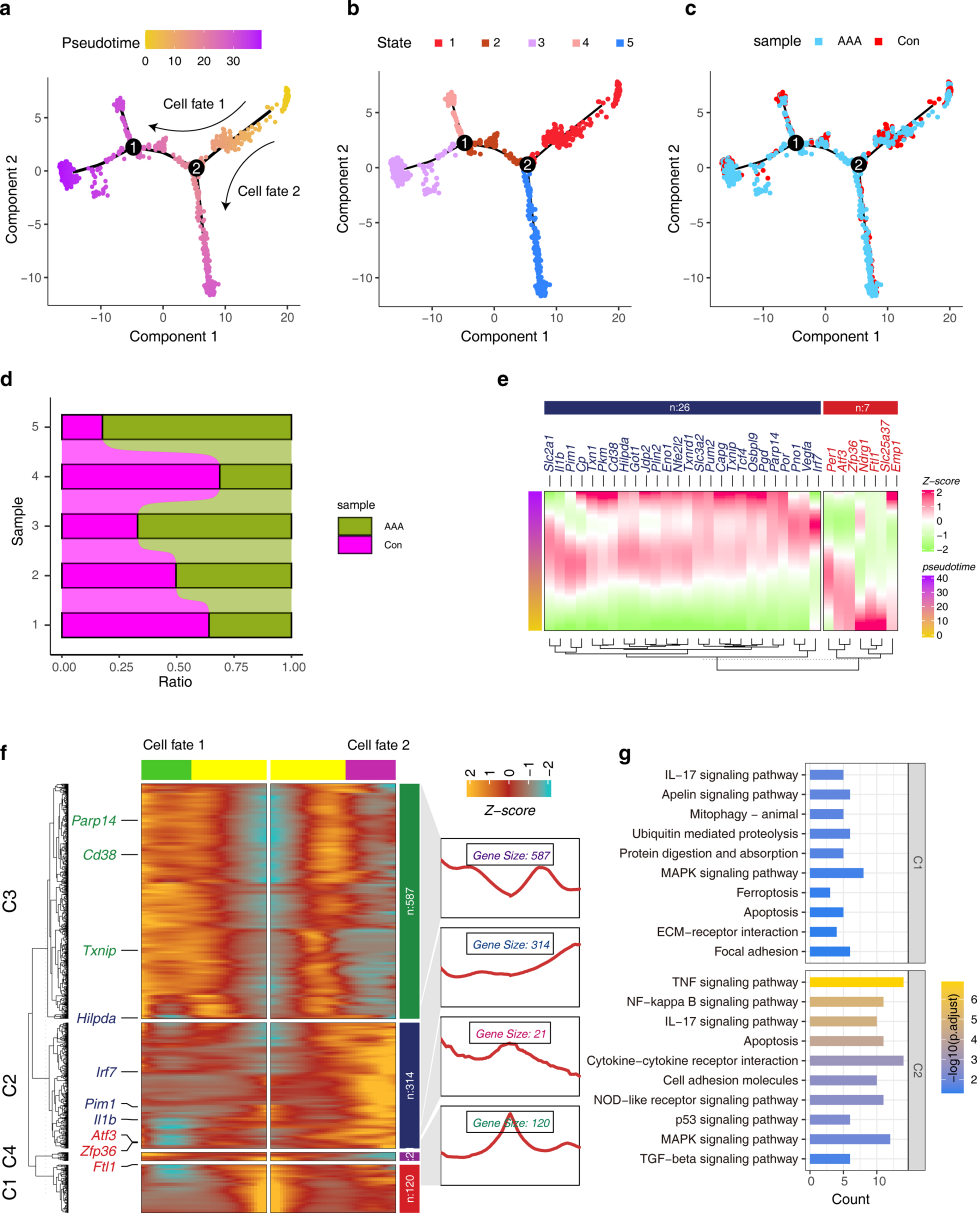
Construction of pseudo-development trajectories of Mo/MF cells. **(A, B)** The pseudo-time trajectories of Mo/MF show different fate trajectories **(A)** and different cell states **(B)**. **(C)** Distribution of different groups under pseudo-time trajectory. **(D)** The stacked bar graphs show the distribution patterns of different groups in different cell states. **(E)** Expression trends of candidate cuproptosis-associated ferroptosis genes under pseudo-temporal trajectories. **(F)** The heat map shows the gene expression trends before and after cell fate 2. **(G)** The KEGG enrichment analysis of gene sets with different patterns in the heatmap.

To investigate whether cuprotosis-associated ferroptosis genes regulate Mo/MF fate decisions, we performed developmental trajectory construction. The results showed that during Mo/MF development, two fate-determination events occurred and all cells were divided into five states (**Figures 6A, B**). Next, we observed the distribution of AAA cells in the trajectory and counted the proportion of AAA cells in different states to determine which cell fate determination period affects the functional state of Mo/MF, that is, to explain the driving factors of AAA (**Figures 6C, D**). The results showed that the proportion of AAA cells was high around the cell fate 2 period, indicating that this stage was driven by AAA. Elucidating the transcriptional dynamics of this fate will help reveal the regulatory mechanism of AAA. Subsequently, we visualized the expression signatures of candidate cuprotosis-associated ferroptosis genes on the developmental trajectory, and the results showed that 33 genes were involved in fate trajectory construction, indicating that ferroptosis is indispensable for AAA progression (**Figure 6E**). Importantly, we explored the transcriptional signatures that regulate cell fate 2 and revealed four distinct sets of genes involved in driving cell fate 2, including several cuprotosis-associated ferroptosis genes, such as *Pim1, Ftl1*, and *Cd38* (**Figure 6F**). We investigated the functional characteristics of genes in different datasets and the results showed that the C1 gene set was involved in the ‘IL17 signaling pathway’, ‘ferroptosis’, and ‘apoptosis’. The C2 gene set was also involved in the’ IL17 signaling pathway’ and ‘apoptosis’ (**Figure 6G**). The above results show the key role of ferroptosis-related genes in the regulation of Mo/MF fate.

### 3.7 Using hdWGCNA to identify candidate cuprotosis-associated ferroptosis genes

**Figure 7:**
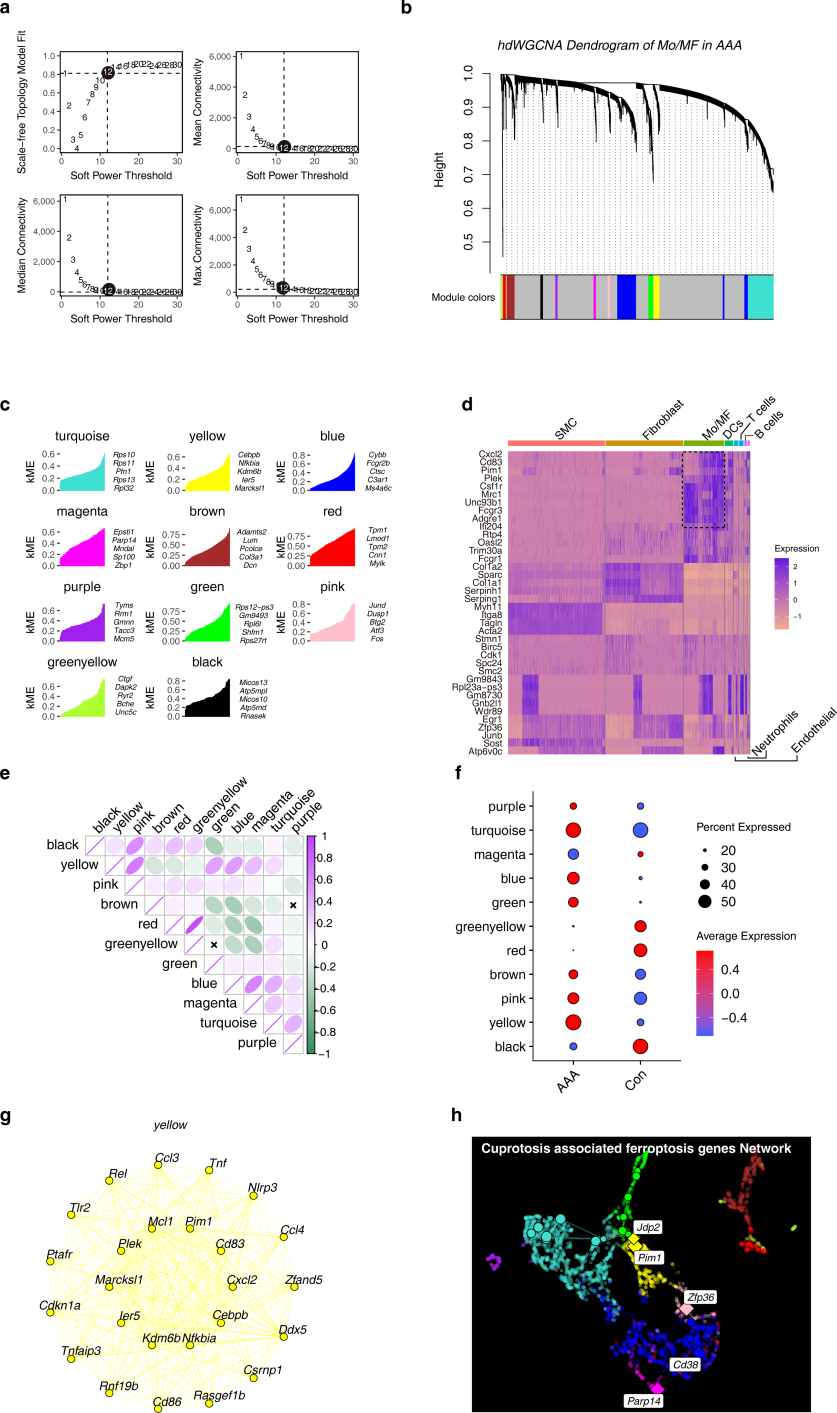
Construction of WGCNA in Mo/MF at the single-cell level. **(A)** Free-scale network topology analysis for different soft threshold powers. The black circle shows the optimal threshold chosen. **(B)** Hierarchical clustering numbers show the modules to which genes belong. **(C)** The genes in each module ranked by kME that iseigengene-based connectivity. **(D)** The heat map shows the expression characteristics of hub genes in different cell types. **(E)** Correlation analysis between different modules. **(F)** The dot plot shows the expression patterns of different modules under different groups. **(G)** Co-expression network of the top 25 genes in the yellow module. **(H)** Co-expression network of cuproptosis-associated ferroptosis genes in Mo/MF.

WGCNA is an analysis algorithm that effectively screens key genes, and we performed hdWGCNA in Mo/MF to identify drivers of AAA. In order to construct a scale-free network, we screened the β value, and the results showed that the most appropriate value was β = 12 (**Figure 7A**). Then, we divide the genes into modules, and the hierarchical clustering numbers show the modules to which different genes belong (**Figure 7B**). 11 modules are obtained, and we use different colors to represent the modules (**Figure 7C**). We further studied the expression characteristics of hub genes within the module and found that these hub genes were cell type specific. In Mo/MF, *Cxcl2, Cd83, Pim1* and other hub genes were highly expressed (**Figure 7D**). Interestingly, we found that different modules have different correlations, such as the yellow module is significantly positively correlated with the pink module, while the red module is negatively correlated with magenta (**Figure 7E**). This result suggests that different modules drive different biological events, and that there is crosstalk and antagonism between different modules. Moreover, we explored the relationship between different modules and different phenotypes. The results showed that the yellow module was significantly overexpressed in AAA (**Figure 7F**). We further observed the hub genes within this module and found that *Pim1, Cd83* and others were highly connected (**Figure 7G**). Of note, we constructed a cuprotosis-associated ferroptosis genes regulatory network and discovered five key factors, including *Pim1, Jdp2, Zfp36, Cd38 and Parp14* (**Figure 7H**).

### 3.8 Transcriptional signature of PBMCs in AAA

**Figure 8:**
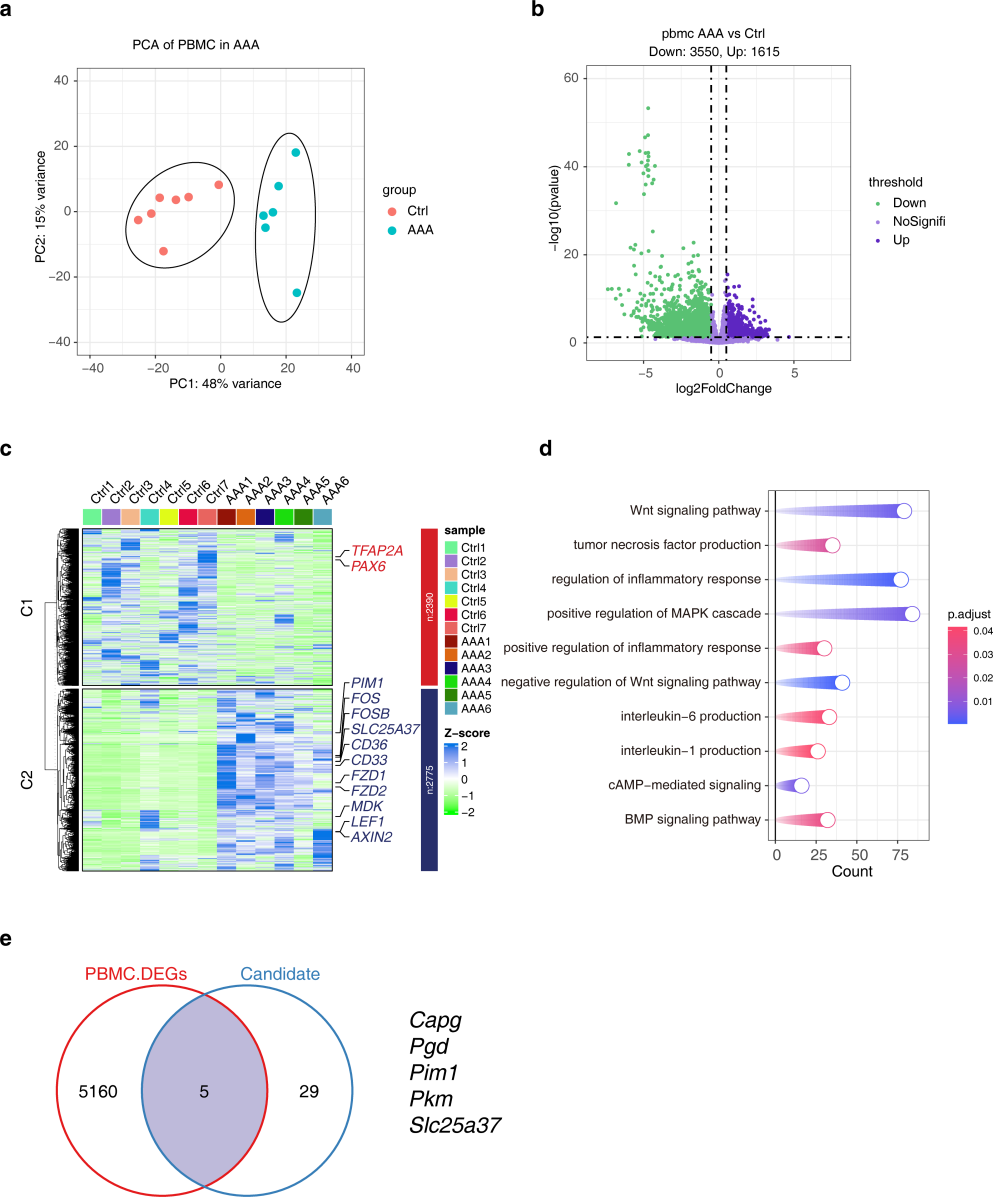
Analysis of transcriptional differences in PBMCs in AAA. **(A)** PCA analysis shows the top2 PCs under different groups. **(B)** The volcano plot shows the distribution of DEGs, and the DEGs screening threshold was |log2FC| > 0.5 and *p* < 0.05.** **(C)** The heat map showing the expression characteristics of DEGs. **(D)** KEGG enrichment results of DEGs. **(E)** The Venn diagram showing the comparative analysis of DEGs and cuproptosis-associated ferroptosis genes. AAA1-6 means different sample.

The main purpose of this study was to screen effective biomarkers of cuprotosis-associated ferroptosis genes. Given the difficulty in manipulating tissues, we sought to consider the characteristics of these candidate genes in PBMCs, which are more easily accessible and manipulatable in the clinic. We included PBMC data from 7 donors and 6 AAA patients, and PCA analysis showed that they had obvious transcriptional distinction (**Figure 8A**). 5,165 DEGs were obtained, and the heat map showed their expression characteristics (**Figures 8B, C**). Functional enrichment analysis showed that DEGs were involved in a large number of inflammatory signaling pathways, such as ‘TNF-α production’, ‘IL-6 production’. ‘IL-1 production’ (**Figure 8D**). Furthermore, we analyzed the relationship between DEGs and uprotosis-associated ferroptosis genes, and the results suggested that they had five overlaps, namely *Capg, Pgd, Pim1, Pkm and Slc25a37* (**Figure 8E**).

### 3.9 Multiple machine learning models combined with bioinformatics to identify key markers

**Figure 9:**
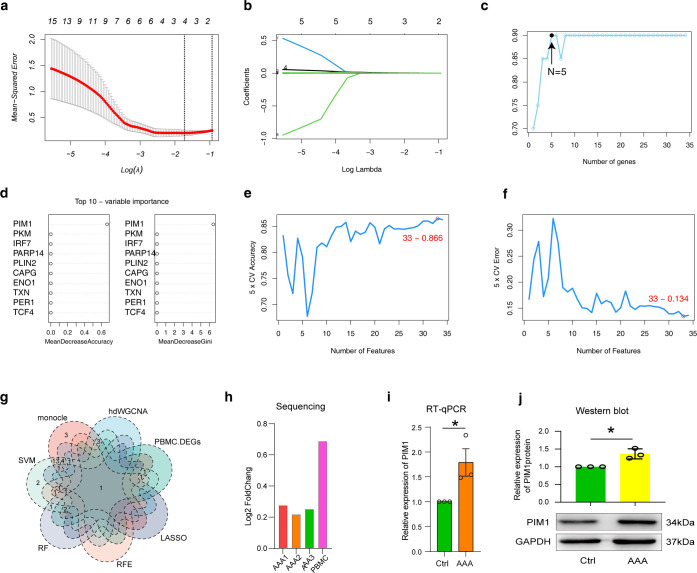
Analysis of transcriptional differences in PBMCs in AAA. **(A)** and **(B)** LASSO regression analysis was performed in PBMC data using candidate cuproptosis-associated ferroptosis genes. The minimum value was defined based on 10-fold cross validation, where the best λ yielded 4 cuproptosis-associated ferroptosis genes **(A)**. Coefficient curves were plotted according to (log λ) sequence and lambda value **(B)**. **(C)** The REF model generated five candidate cuproptosis-associated ferroptosis genes. **(D)** The top 10 cuproptosis-associated ferroptosis genes generated by RF model evaluation. **(E, F)** Under the SVM model, the line graph shows the accuracy **(E)** and error of different genes **(F)**. **(G)** The Venn diagram shows the comparative analysis of candidate cuproptosis-associated ferroptosis genes obtained by screening with different bioinformatics algorithms and machine models. **(H–J)** The expression characteristics of PIM1 were examined by sequencing **(H)**, RT-qPCR **(I)** and western blot **(J)**. AAA1 means CaCl_2_-induced model, AAA2 means elastase-induced model and AAA3 means Ang II-induced model.

Machine learning models are a good method for selecting biomarkers. Next, we used four machine models, including LASSO, REF, RF and SVM, to select biomarkers in a PBMC transcriptome data from AAA. We used 34 cuproptosis associated ferroptosis genes as features (**Figure 5D**), and used AAA and normal as predictive target variables to build the model. According to the LASSO model, four genes were identified as candidate genes, including *PIM1, PLIN2, TXNRD1 and NDRG1* (**Figures 9A, B**). REF obtained five candidate genes, including *PIM1*, *PLIN2, VEGFA, NDRG1 and SLC25A37* (**Figure 9C**). RF pick out ten candidates, including *PIM1, PKM, IRF7, PARP14, PLIN2, CAPG, ENO1, TXN, PER1 and TCF4* (**Figure 9D**). The accuracy and error rate in the SVM model showed that 33 genes were the most suitable (**Figures 9E, F**). Significantly, we considered all the data, including three bioinformatics analysis methods and four machine learning algorithms, and finally obtained a candidate gene, namely PIM1 (**Figure 9G**). As expected, PIM1 was significantly overexpressed in AAA in all sequencing data, which strongly suggested the potential of this gene as a biomarker (**Figure 9H**). To verify the expression characteristics of PIM1 in patient PBMCs, we used RT-qPCR and protein immunoblotting to verify it at the RNA and protein levels. Excitingly, our experimental results suggest that PIM1 is significantly overexpressed in AAA, both at the RNA and protein levels (**Figures 9I, J**).

## 4 Discussion

Currently, the only treatment options for AAA are surgery and endovascular repair. Unfortunately, conservative treatment options are lacking (1, 32). Emerging evidence suggests that two newly discovered programmed cell death, cuproptosis and ferroptosis, play crucial roles in the progression of AAA and show promise as effective future treatments for AAA (14, 33–35). However, studies exploring genes associated with cuprotosis and ferroptosis in AAA are lacking. Therefore, it is crucial to focus on potential prognostic studies on the link between cuprotosis and ferroptosis in AAA.

To select appropriate cell types for research, we first analyzed the differences in cell types in different AAA models. Consistent with previous studies, we found that SMCs were significantly reduced in AAA, which can be explained by the pathological characteristics of extensive apoptosis of SMCs in AAA (15–17, 36). The AUGUR algorithm suggests that Mo/MF cells are most affected by AAA, which may be related to the inflammatory response in AAA (2, 5). The functional enrichment results of DEGs in different models suggested that ferroptosis was significantly enriched, and the common DEGs of different models also showed enrichment for ferroptosis, which suggests that ferroptosis plays a role in driving AAA (33). Based on the above reasons, we studied cuprotosis-associated ferroptosis gene features in Mo/MF.

The construction of pseudo-temporal developmental trajectories provides a means to study cell fate determination (26). Our results found that among the genes driving Mo/MF changes in AAA, there are shadows of uprotosis-associated ferroptosis genes. Consistent with previous studies, our results suggest that ferroptosis drives AAA progression and that inflammatory responses occur extensively in Mo/MF at the AAA cell fate stage, which is characterized by the enrichment of the TNF-α signaling pathway. (5, 34, 37). The rise of hdWGCNA has broadened the perspective of single-cell data analysis and provided the possibility for better mining of candidate genes (29). A total of five candidate genes were obtained, including *Pim1, Jdp2, Zfp36, Cd38 and Parp14.* Currently, studies have reported that the expression level of CD38 is positively correlated with the diameter of the abdominal aorta and that the lack of CD38 in SMCs can inhibit the progression of AAA (38, 39).

Back to our topic, screening for effective AAA biological targets, considering the difficulty in obtaining samples in the clinic, we attempted to study the characteristics of candidate genes in PBMCs to more quickly advance our results to clinical translation.

Monocytes in PBMCs, as the source of MF, can reflect the characteristics of MF in tissues to a certain extent (2, 4, 5). Consistent with previous reports, we found that PBMCs in AAA underwent a substantial inflammatory response (18, 37). Our cross-analysis revealed five candidate genes, including *Capg, Pgd, Pim1, Pkm and Slc25a37*. Previous studies have reported that different expression forms of PKM affect the progression of AAA (40). These data suggest that the genes we selected have the potential to become biomarkers. Machine learning models have been widely used in predicting biomarkers and building prognostic models (41, 42). Four different machine learning methods, LASSO, RFE, RF and SVM, were selected to predict AAA markers, with candidate genes ranging from 4 to 33. This result shows that different machine learning models have different predictive effects (43).

A single bioinformatics analysis algorithm and machine learning model is not sufficient to screen effective biomarkers. We conducted a comprehensive comparison of all analysis methods, and excitingly, we obtained a potential biomarker, PIM1 (Pim-1 proto-oncogene, serine/threonine kinase), which was obtained in all analyses. PIM1 is a proto-oncogene that encodes a serine/threonine kinase that is primarily involved in cell cycle progression, apoptosis, transcriptional activation, and general signal transduction pathways (44, 45). Due to its critical functions, it has been extensively studied as a therapeutic target in cancer, but there is a lack of reports on its research in AAA. Interestingly, a recent report showed that it could regulate macrophage infiltration and polarization in the tumor microenvironment to enhance anti-PD-1 therapy response (45), which showed its effect on macrophages and also suggested that it might affect macrophages in AAA and thus regulate AAA progression. Indeed, PIM1 has been reported as a marker for pulmonary hypertension (46). These results strongly suggest that PIM1 may serve as an effective biomarker for AAA. To verify it, we characterized the gene and protein expression patterns of AAA in PBMCs of AAA patients. As expected, PIM1 was significantly upregulated in AAA. In a conclusion. PIM1 as a novel biomarker associated with cuproptosis/ferroptosis in AAA was highlighted.

We have to admit that the current study also has some limitations. This study is a hypothesis-driven study based on scRNA-seq or bulk RNA-seq data. Although we have verified the expression pattern of PIM1 in AAA through experimental schemes and confirmed it as an effective biomarker. However, its biological function in AAA has not yet been determined in this study. In future studies, the study of the biological function of PIM1 needs to be strengthened.

## 5 Conclusions

In summary, the combined results of our bioinformatics and machine learning models highlighted PIM1 as a valid biomarker for AAA, which was validated by RT-qPCR and western blot.

## Data Availability

This study uses public database datasets, including GSE152583 (https://www.ncbi.nlm.nih.gov/geo/query/acc.cgi?acc=GSE152583), GSE164678 (https://www.ncbi.nlm.nih.gov/geo/query/acc.cgi?acc=GSE164678) and GSE221789 (https://www.ncbi.nlm.nih.gov/geo/query/acc.cgi?acc=GSE221789). No new codes were generated in this study, and all codes can be found in the relevant software guidance documents.

## Supplementary material

Supplementary Figure 1 Supporting for **Figure 1**. **(A, B)** UMAP plots show the distribution of samples **(A)** and seurat_clusters **(B)** under the three AAA models. **(C)** UMAP profiles of annotated cell types for each sample.

Supplementary Figure 2 Supporting for **Figure 2**. **(A)** The bubble chart shows the differential communication signals between Mo/MF and endothelial in different groups. The left side shows the up-regulated signals in AAA, and the right side shows the down-regulated signals.
