# Supplementary figures and images for: Integration of bulk/scRNA-seq and multiple machine learning algorithms identifies PIM1 as a biomarker associated with cuproptosis and ferroptosis in abdominal aortic aneurysm
Source: Front Immunol. 2024 Dec 11;15:1486209. doi: 10.3389/fimmu.2024.1486209 (PMC11668634; doi:10.3389/fimmu.2024.1486209)

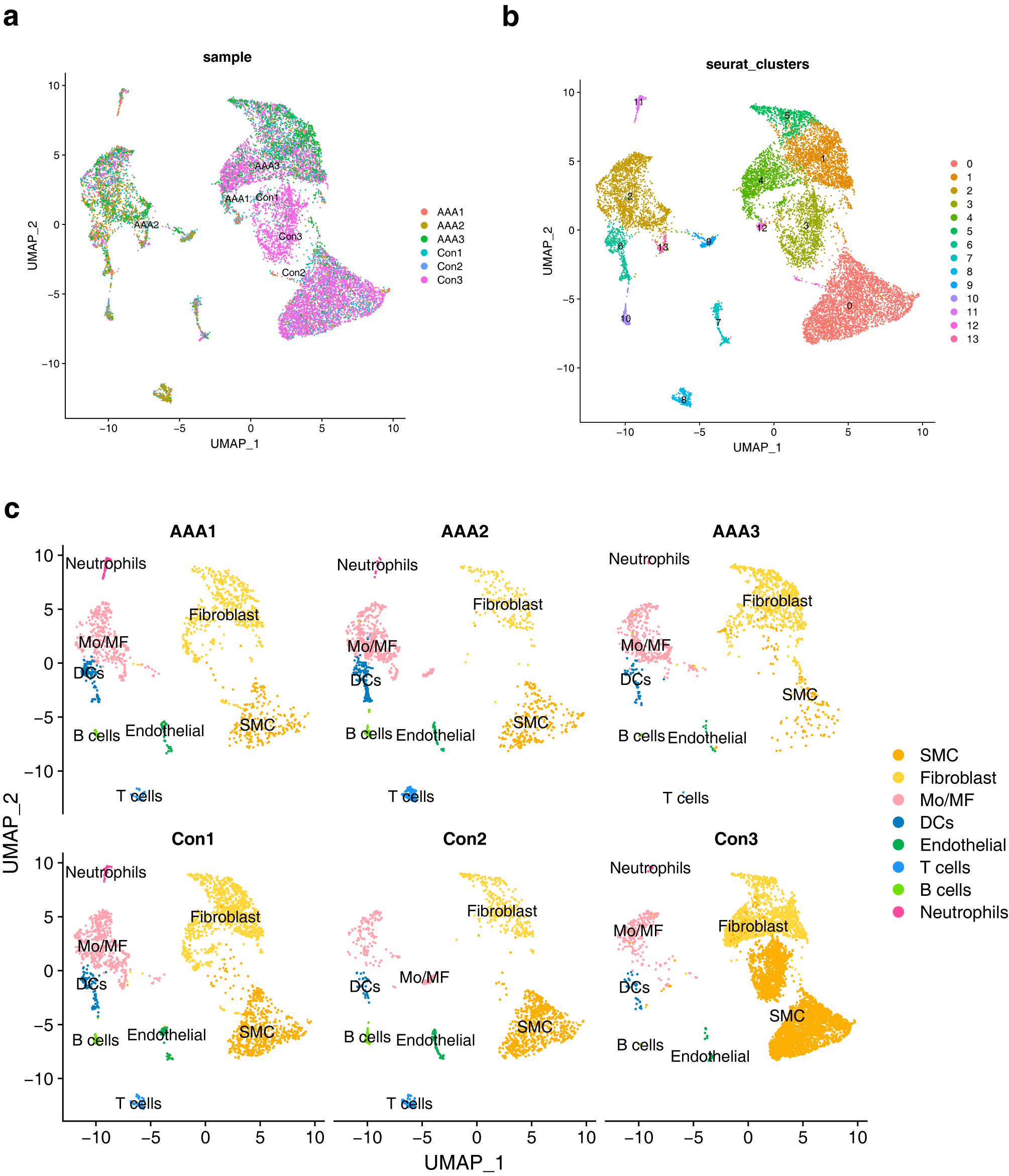

Supplement: Supplementary Figure 1 — Supporting for Figure 1 . (A, B) UMAP plots show the distribution of samples (A) and seurat_clusters (B) under the three AAA models. (C) UMAP profiles of annotated cell types for each sample. [file Image1.tif]

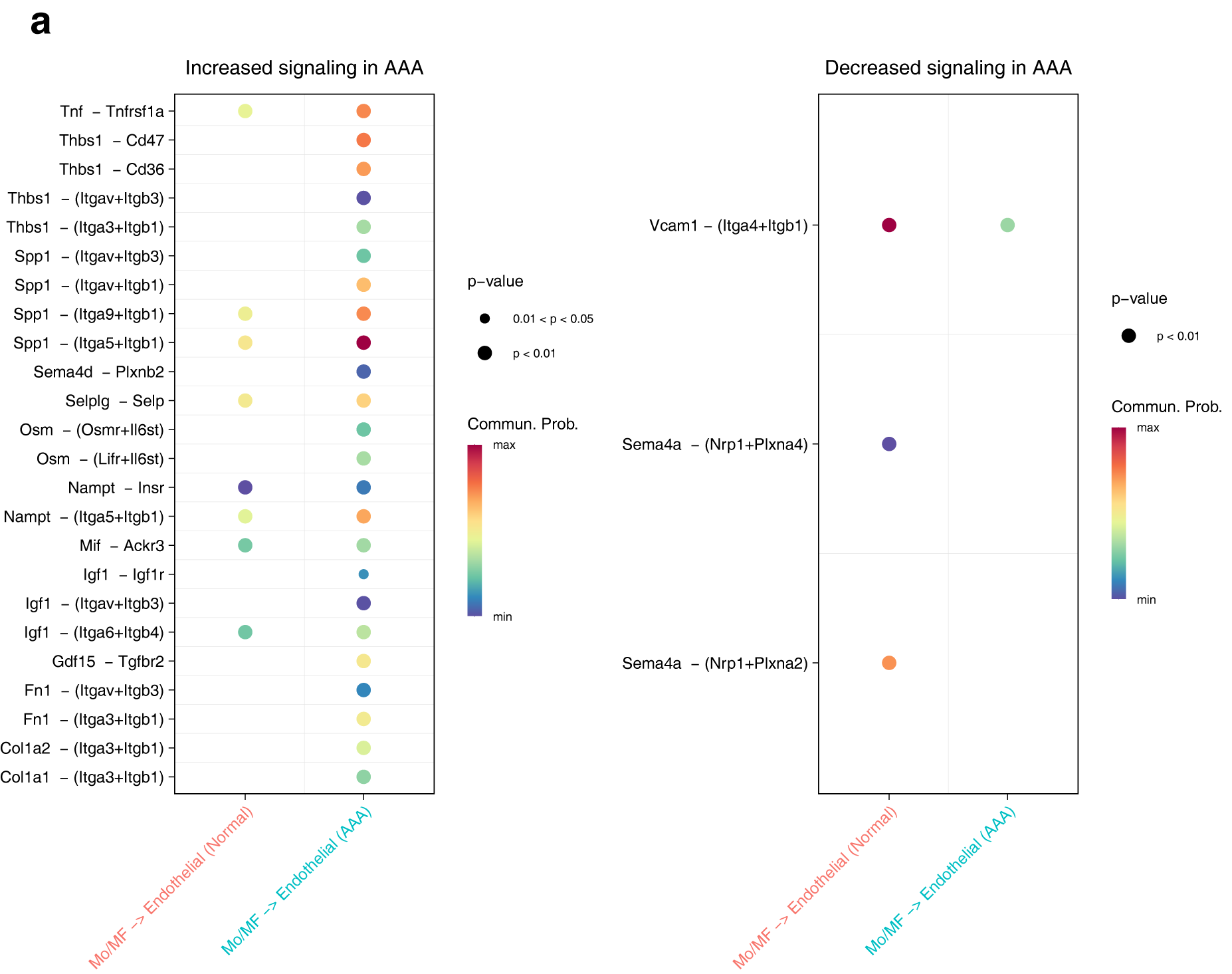

Supplement: Supplementary Figure 2 — Supporting for Figure 2 . (A) The bubble chart shows the differential communication signals between Mo/MF and endothelial in different groups. The left side shows the up-regulated signals in AAA, and the right side shows the down-regulated signals. [file Image2.tif]
