# Supplementary material for: Integration of bulk/scRNA-seq and multiple machine learning algorithms identifies PIM1 as a biomarker associated with cuproptosis and ferroptosis in abdominal aortic aneurysm
Source: Front Immunol. 2024 Dec 11;15:1486209. doi: 10.3389/fimmu.2024.1486209 (PMC11668634; doi:10.3389/fimmu.2024.1486209)
Supplement: Supplementary file 3 [file Table1.docx]

**TableS1. The information of data collection.**

| GEO ID | Species | Sequencing Type | Tisusue (model) | Sample size | Main analysis |
| --- | --- | --- | --- | --- | --- |
| GSE164678 | Mouse | Single-cell | Abdominal aortas  (CaCl_2_-induced) | 3933 cells,  (2513 Ctrl, 1420 AAA) | Expression analysis, pseudo-chronological trajectory, and hdWGCNA |
| GSE152583 | Mouse | Single-cell | Abdominal aortas  (elastase-induced) | 2948 cells,  (1542 Ctrl, 1406 AAA) |  |
| GSE221789 | Mouse | Single-cell | Abdominal aortas  (Ang II-induced) | 7208 cells,  (5743 Ctrl, 1465 AAA) |  |
| *Ref: Int. J. Mol. Sci., 22(6), 3200.* | Human | Bulk RNA | PBMC | 13 sampels,  (7 Ctrl, 6 AAA) | Expression analysis and machine learning |
