# Supplementary material for: Integration of bulk/scRNA-seq and multiple machine learning algorithms identifies PIM1 as a biomarker associated with cuproptosis and ferroptosis in abdominal aortic aneurysm
Source: Front Immunol. 2024 Dec 11;15:1486209. doi: 10.3389/fimmu.2024.1486209 (PMC11668634; doi:10.3389/fimmu.2024.1486209)
Supplement: Supplementary file 4 [file Table2.docx]

| GENE | Forward Primer | Reverse Primer |
| --- | --- | --- |
| PIM1 | GGCTCGGTCTACTCAGGCA | GGAAATCCGGTCCTTCTCCAC |
| GAPDH | ACAACTTTGGTATCGTGGAAGG | GCCATCACGCCACAGTTTC |

**TableS2. The information of primer.**
